# A Psychoeducational Intervention in Prenatal Classes: Positive Effects on Anxiety, Self-Efficacy, and Temporal Focus in Birth Attendants

**DOI:** 10.3390/ijerph19137904

**Published:** 2022-06-28

**Authors:** Pierluigi Diotaiuti, Giuseppe Valente, Stefania Mancone, Lavinia Falese, Stefano Corrado, Thais Cristina Siqueira, Alexandro Andrade

**Affiliations:** 1 Department of Human Sciences, Society and Health, University of Cassino and Southern Lazio, 03043 Cassino, Italy; giuseppe.valente@unicas.it (G.V.); s.mancone@unicas.it (S.M.); l.falese@unicas.it (L.F.); stefano.corrado@unicas.it (S.C.); 2 Health and Sports Science Center, Department of Physical Education, CEFID, Santa Catarina State University, Florianópolis 88035-901, Brazil; thais.siqueira@udesc.br (T.C.S.); alexandro.andrade.phd@gmail.com (A.A.)

**Keywords:** prenatal education, pregnancy, anxiety, self-efficacy, time orientation, mental health

## Abstract

Background: Previous studies have reported associations between high maternal anxiety, temporal perceptions during pregnancy, and a poor sense of self-efficacy. One type of anxiety expecting mothers experience is associated with childbirth, which previous studies have shown can be reduced by antenatal training. Recent contributions have pointed out that current prenatal courses, while providing important and useful knowledge, do not devote sufficient content to the mental health of the parturient and to the psychological issues that can arise before and after the birth. Methods: In total, 80 pregnant women were provided with a special prepartum course in which ample space was devoted to topics such as maternal mental health, parenting skills and couple relationship, relaxation techniques, and assertiveness. Perception of threat, state anxiety, temporal focus, needs and expectations, and self-efficacy were assessed by comparing this psychoeducational intervention group with a traditional antenatal course group (*n* = 80), and a control group (*n* = 80). Two-way mixed ANOVAS (3 × 2) were performed for each dependent variable considered, including the time variable (pre-course–post-course) as a factor within the participants and the group variable as a factor between the subjects. Results: The psychoeducational intervention actually induced significant and positive changes primarily on four dimensions: state anxiety, perceived self-efficacy, the need for information, and reassurance of the pregnant women who participated in this trial. Conclusions: The study suggests improving the quality of prenatal classes by paying particular attention to the content and communication used within the group, in order to gratify at the highest level, the need for information, reassurance, and sharing that characterize the parturient’s request for support. The evidence collected recommends further replicating the intervention protocol described in order to improve the psychophysical well-being of women in a delicate moment such as pregnancy and preparation for childbirth, but especially in terms of the prevention and containment of the risks of psychological distress that currently affect a significant number of women after childbirth.

## 1. Introduction

Epidemiological studies report that the presence of anxiety disorders is particularly common in the female population, affecting about 30% of women in their lifetime [1]. Anxiety often occurs in comorbidity with depressive disorders [2], and, recently, a growing number of studies have shown that it often tends to accompany depression in its perinatal period, which is a particularly dynamic phase, in which there are conflicting thoughts and moods, which are pleasant and worrying at the same time [3,4]. It is normal for pregnant women or new mothers to report symptoms of anxiety and worry regarding the possibility of giving birth to a baby with abnormalities; having complications during pregnancy or during birth; or concerns about one’s ability to care for the baby, including breastfeeding and calming the baby when he or she cries; concerns may also relate to changes in one’s body, relationship with a partner, job performance, or any financial issues. According to some scholars, although anxiety and depression are more common during pregnancy than in the postpartum period, the postpartum symptoms have received more scholarly and clinical attention [5,6,7,8]. Pregnancy is in fact one of the most important moments in a woman’s life, as it brings with it many changes, not only in the physical, but also in the psychological and social aspects [9]. Becoming a mother is an overwhelming life experience that often generates stress [10,11,12,13,14,15,16,17].

Fear of the unknown, feelings of uprooting, and daily problems associated with physical and hormonal changes, typical of the gestation period, make this period more conducive to a high level of anxiety [18]. Anxiety is a construct also linked to time. In fact, future-oriented thinking has been considered a central feature of anxiety. Future-oriented anxious and depressive thought patterns are largely based on recovery bias [19]. However, a fundamental premise underlying temporal research is the idea that people differ in their perceptions of the past, present, and future [20,21,22]. 

That is, despite the unidirectional progression of time, individuals can mentally move back and forth between the “stabilized past” and the “obscure preconceptions of what lies ahead” [23,24]. Numerous empirical studies showed that high maternal anxiety, and temporal perceptions during pregnancy diminish the sense of self-efficacy and can influence the outcomes of fetal development and subsequently of the child [25]. 

Time attention is important because thinking about the past, present, and future influences current attitudes, decisions, and behaviors, as evidenced by research on goal setting, motivation, and performance [26,27,28,29,30]; self-regulation [31,32]; sensitization [33]; affect [34]; and strategic choice [35,36,37]. As women in labor are psychologically committed to controlling their fear and anxieties about the changes and unexpected events involved in childbirth, it seemed useful to assess the prevailing temporal focus before and after the course. Expectations regarding trends were that the future focus would prevail before the course, and that the present focus would increase after the prebirth course had ended. The prevalence of orientation on the future focus of parturients has already been observed in a recent study by Li and Cao [38]. The relationship between time perspective and coping mechanisms was investigated in Bolotova and Hachaturova [39], while the Oyanadel and Buela-Casal clinical sample study [40] highlighted how the time perspective characterizes the psychopathology and well-being of individuals with psychiatric problems. More specifically, a relevant study by Åström et al. [41] has already examined the time perspective in persons with anxiety. Papastamatelou et al. [42] argued that temporal orientation could be a predictor of perceived anxiety and stress and also suggested the importance of further studies to assess whether a change of time perspective can contribute to the treatment of generalized anxiety and to the reduction of perceived levels of stress, and thus alleviate two negative factors responsible for reduced well-being. Zimbardo et al. [43] prefigured a specific therapeutic approach on PTSDs based on the manipulation of the patient’s time perspective.

Pregnant women with high levels of stress and anxiety present a high risk of adverse perinatal outcomes [44]. Anxiety during pregnancy is associated with prematurity, low birth weight, and restriction of fetal growth, and types of alterations in the child’s immune system which, in turn, are risk factors for the child’s cognitive and social development [45,46,47]. It has also been shown that depressive and anxiety symptoms in pregnant women have a negative impact on the development of the woman’s relationship with the fetus, the newborn and, subsequently, with the child [48,49].

A pregnant woman may have anxieties related to the fear of the child’s health but also to herself [50,51]. Added to this is the fear of childbirth that represents a stressful event as unpredictable, uncontrolled, and inevitable [52]. While women may have expectations for labor, as regards childbirth they do not know exactly when it will happen, how much pain they will suffer, and whether the outcome will be positive or whether the birth will be planned [53]. 

The high degree of anxiety associated with physical changes in the gestational period can also alter the quality of sleep of pregnant women, influencing their performance in activities, in addition to resulting in confusion, generating feelings of uncertainty and the inability to control emotions, and further elevating the degree of anxiety [54,55,56]. 

A significant tool for anxiety management could be antenatal training, which takes place through specific courses [57]. There are many doubts about the value of antenatal training for the management of pain and stress during childbirth [58]. However, in recent years, numerous studies have shown that antenatal training is often a good way to remove or alleviate fears associated with pregnancy and childbirth because it provides knowledge and skills to pregnant women and the family [59,60]. Further studies have shown that antenatal training allows a greater knowledge of the birth path, increases the degree of birth satisfaction, and strengthens the self-efficacy linked to childbirth and the sense of control during birth [61,62,63,64,65,66,67]. 

However, researchers have paid little attention to the content of antenatal lessons. Studies that investigated course contents occurred mainly retrospectively after childbirth, when parents realized that the lessons did not address key issues. As a result, there is a need to obtain information on the issues raised by parents during antenatal classes [68,69]. Self-efficacy, parental emotional health, parenting skills, and the couple’s relationship are often among the many factors that are not usually addressed during antenatal classes [70,71]. 

Having acknowledged these critical aspects raised by the recent literature on the content of the courses that are traditionally provided to prepare women for childbirth, the objectives of the present study were: (1) to present a psychoeducational intervention carried out with parturients for whom a special program of content was prepared, with ample space devoted to topics such as maternal mental health, parenting skills and the couple’s relationship, relaxation techniques, assertiveness, construction of a realistic model of the mother, and cognitive restructuring work; (2) comparing trends in variables such as anxiety, perceived self-efficacy, and temporal orientation in this group vs. a group that attended a traditional prepartum course (seven meetings without the psychological content) and vs. a group of parturients who did not attend any course (control). The hypothesis was that the planned course would primarily produce a beneficial effect on perceived self-efficacy with the reduction of anxiety in the parturient, in comparison to those who attended the traditional course and those who did not attend any prebirth classes. Furthermore, in the control group that had not attended a prenatal course, there would be an increase in the need for information, reassurance and sharing, as well as in anxiety, and a decrease in the perceived self-efficacy levels. 

## 2. Materials and Methods

### 2.1. Study Design

This is a non-randomized quasi-experimental study.

### 2.2. Sample

The sample was non-probabilistic intentional. The study involved a total of 240 pregnant Italian women. Among these, 80 took part in a traditional prebirth course organized by a local health district of the Lazio Region; 80 took part in a special prebirth course enriched with psychological content and planned together with the group of researchers from the University of Cassino; and 80 did not attend any courses but gave their availability for the measurement of some psychological variables during their pregnancy, thus acting as a control group in the study. Regarding recruitment, the first two groups were established through the activation of the local health district and composed by the randomization of participants once they had declared their adherence to the project; whereas the control group was obtained through a free invitation to participate mediated by university students who had relatives or friends who were pregnant during the period in which the study was planned. Thus, participation was free and voluntary and did not involve any form of reward. Although the research design with three groups and two course conditions and one control condition allowed for a pre–post comparison even with 33 participants (G*power estimate), thus 11 per group, it was decided to extend the sample composition per group to 80, both to have more robust results, and considering the high rate of voluntary adherence to the project shown by the parturients followed by the territorial health unit. The inclusion criteria for taking part in the study were to be in the condition of first pregnancy, not to have ongoing psychiatric pathologies (depression, personality disorders, psychosis), and not to be under related pharmacological and/or psychotherapeutic treatment. At the beginning of the course, all the women were on average in the 25th week of gestation (SD = 2.71; range 18–31) and had a mean age of 32.24 years (SD = 3.88; interval 24–41). In relation to their education, 2% had a middle school diploma, 40.2% a high school diploma, 46.1% a university degree, 11.8% a postgraduate degree; profession: 21.6% unemployed, 2% factory worker, 51% employed, 17.6% self-employed, 7.8% student; marital status: 4% single, 25.5% cohabiting, 70.6% married. The comparability of the three groups was verified by testing for non-significant differences in their internal composition with respect to the variables of area of residence, education, occupation, and marital status (chi-square test, *p* > 0.05). All participating women had previously released the informed consent for the collection of information in an anonymous form aimed at scientific analyses in aggregate form. 

### 2.3. Procedures

A survey was carried out through the use of an ad hoc built questionnaire, at the beginning of the prebirth course and at the end of it two months later. The control group was also administered the questionnaire for the first time when the women were around 25 weeks of gestation and the second time two months later. The traditional course was held once a week and lasted about two hours, alternately conducted by the members of the team, composed of a gynecologist, a social assistant, a midwife, and a nurse. In addition to the introductory meeting, it included seven thematic meetings dealing with typical topics of pregnancy and childbirth (hygiene in pregnancy, labor, childbirth, umbilical cord donation, breastfeeding, newborn care, childhood vaccination, pelvic floor rehabilitation). Instead, the “enriched course” was structured in two weekly meetings. In addition to the number of meetings and content common to the traditional course, eight additional meetings were introduced and were led by a psychologist. Here to follow is a description of the program administered: *First* *meeting*. Women were informed about the importance of psychological well-being and mental health problems that can occur during pregnancy and after childbirth, as well as their impact on both the mother’s health and the child’s development and on life as a couple. They were asked what expectations they had of motherhood, and the meeting was essentially psychoeducational, normalizing the possibility of not feeling well in such a demanding moment of life, showing the impact that thoughts have on emotions and behavior. *Second meeting*. Future mothers were asked to reflect on their daily routine and the possibility of introducing pleasant activities. A list of suggestions of pleasant activities was distributed and the women were asked to introduce them at least once a day. *Third meeting*. They were taught Jacobson’s progressive muscular relaxation and they tried to identify [72] a “stress-eliminator” that would be quick, easy to apply, and on hand in case of need (mini mental holiday, positive self-affirmation, phone call to a friend). *Fourth meeting*. Assertiveness was dealt with, proposing a set of communication techniques that teach how to express one’s needs correctly, without falling into aggressive or, as often happens, passive attitudes [73]. *Fifth meeting*. Using the genogram, the women were asked to reflect on the image of “mother” that they would like to be, and on the one that is strongly influenced by the model inherited from their own family, by their husband, and by images proposed by the mass media. *Sixth meeting*. Beck’s triad [74] on thoughts/emotions/behavior was once again discussed, inviting the women to take time to write down the thoughts that appear during the day, analyzing them critically and teaching the women to replace them with more useful and realistic thoughts. *Seventh meeting*. Anxiety and fear related to childbirth were discussed in depth and some coping strategies along with techniques to defuse pain perception were illustrated [75]. *Eighth meeting*. The acquired skills were summarized by helping to fill in a personalized form detailing the basic steps that characterized the course: introduction of pleasant activities, organization of the day, relaxation techniques, assertiveness, construction of a realistic mother model, and cognitive restructuring work. The women were also invited to write down short-, medium-, and long-term goals as an incentive to continue the improvement process. Finally, the tests compiled in the initial phase were administered again. In addition, respiratory autogenic training (RAT), a method inspired by Schultz’s autogenic training [76,77] and adapted to the specific needs of the pregnant woman [38], was also applied in each meeting. Before the administration, each participant was given voluntary informed consent with a description of the questionnaire and reassurance on the scientific and aggregate use of the data provided, in accordance with the Declaration of Helsinki. The protocol was approved by the Institutional Review Board of the University of Cassino and Southern Lazio.

### 2.4. Instruments

A general questionnaire was administered covering a sociodemographic and a psychometric section. The latter included: a scale for the detection of the subjective perception of threat and state anxiety, the *Endler Multidimensional Anxiety Scales* (Emas) [78]; the *Temporal Focus Scale* (TFS) [79]; finally, a scale built specifically for the detection of the needs and expectations that eventually motivated the women to participate in the course, namely, the *Scale of Needs and Expectations of the Parturient* (SNEP). The conversations carried out in the past with other groups of pregnant women had highlighted three groups of needs which were particularly felt in the condition of pregnancy: the need to receive information (that is precise, useful, reliable); the need to receive reassurance (on the good progress of the pregnancy and on the outcome of the birth); the need to share (the new and transforming experience that is being lived). To these was added the need for self-efficacy (i.e., feeling capable of managing what is happening and what might happen). An up-to-date reference on the information-seeking behavior and the sharing need of the parturient can be found in Lu et al. [80], while reassurance seeking as a strategy to cope with the fear of childbirth was already mentioned in Rondung et al.’s review [81].

Since at the moment there is no Italian validation of a specific instrument for the evaluation of perinatal anxiety, similarly to other previous studies [82,83] we used an instrument to measure general anxiety, which, compared to the more widespread state-trait anxiety inventory (STAI), includes the measurement of the subjective perception of threat related to the situation, which seemed particularly appropriate in the case of childbirth. Thus, the Emas-P and Emas-S Scales were used. Emas-P reflects the measure of subjective perception of the type and degree of threat evoked in a specific situation. It includes 5 items with a Likert scale from 1 (not at all) to 5 (very much) (e.g., “Imagine a situation in which you are evaluated, judged, observed: indicate the degree to which you feel involved”); Cronbach’s alpha = 0.92; 95% CI [0.93–0.97]; McDonald’omega = 0.95; 95% CI [0.93–0.97]. Emas-S indicates a transient emotional state in reference to a particular situation measuring the emotional component of the autonomic nervous system and the cognitive concern through 20 items. Each item is evaluated on a 5-point intensity scale, from 1 (not at all) to 5 (very much) (e.g., “I feel very worried”); Cronbach’s alpha = 0.95; 95% CI 0.93–0.97; McDonald’omega = 0.95; 95% CI [0.93–0.97]. 

*TFS* indicates the measure of persistent orientation of the attention of the individual towards the three main temporal dimensions: past, present, and future. It consists of 10 items divided into three subscales: past focus (4 items; e.g., “I reflect on what has happened in my life”, Cronbach’s alpha = 0.79; 95% CI 0.72–0.85; McDonald’omega = 0.81; 95% CI 0.71–0.87); current focus (3 items; e.g., “I focus on what is currently happening in my life”, Cronbach’s alpha = 0.70; 95% CI 0.58–0.78; McDonald’omega = 0.70; 95% CI 0.59–0.79); future focus (3 items; e.g., “I think about what my future has in store”, Cronbach’s alpha = 0.80; 95% CI 0.73–0.86; McDonald’omega = 0.80; 95% CI 0.71–0.87). Each item is evaluated on a 7-point intensity scale, from 1 (never) to 7 (always). Overall reliability of Cronbach’s alpha scale 0.74; 95% CI 0.67–0.81; McDonald’omega = 0.76; 95% CI 0.68–0.82.

The *SNEP*, built ad hoc as a self-report tool for the present study, includes: the *Perceived Need for Information* (3 items; e.g., “How important is the need that you feel at this time to obtain reliable information by experienced personnel?”; Cronbach’s alpha = 0.95; 95% CI 0.93–0.97; McDonald’omega = 0.95; 95% CI 0.93–0.97); the *Perceived Need for Reassurance* (3 items; e.g., “How important is the need that you feel at this time to feel reassured about the success of the birth?”; Cronbach’s alpha = 0.87; 95% CI 0.82–0.91; McDonald’omega = 0.87; 95% CI 0.81–0.92) the *Perceived Need for Sharing* (5 items; e.g., “How important is the need that you feel at this time to share your fears about childbirth with other women?”; Cronbach’s alpha = 0.88; 95% CI 0.84–0.92; McDonald’omega = 0.89; 95% CI 0.84–0.92); and perceived self-efficacy (3 items; e.g., “How much do you feel able to handle the situation right now?”; Cronbach’s alpha = 0.75; 95% CI 0.66–0.82; McDonald’omega = 0.76; 95% CI 0.61–0.85). Each item was evaluated on a 5-point intensity scale, from 1 (not at all) to 5 (very much). The exploratory EFA (maximum likelihood) with Promax rotation indicated a four-factor structure with positive determinant, KMO (Kaiser–Meyer–Olkin test) = 0.822, significant Bartlett test, overall reliability of Cronbach’s alpha scale 0.83; 95% CI 0.78–0.87; McDonald’omega = 0.85; 95% CI 0.79–0.89. 

### 2.5. Statistical Analysis

For the statistical analyses, we used the package SPSS v. 26 for the verification of the univariate and multivariate hypotheses, the assessment of internal consistency of the scales through Cronbach’s raw coefficient, the exploratory factor analysis (EFA) with maximum likelihood (ML) and Promax rotation for the SNEP scale; JASP 0.12.2 software was used to calculate the McDonald coefficient of internal consistency. In order to evaluate whether the program of psychological area content had significant effects on the variables of anxiety, self-efficacy, and time orientation, and perceived needs compared with the traditional course and the control group, an analysis of variance with a mixed factorial model was established, which included the time variable (pre-course–post-course) as a factor within the participants and the group variable as a factor between the subjects. Therefore, correspondent two-way ANOVA mixes (3 × 2) were performed for each dependent variable considered. Following Cohen [84], partial Eta squared was the measure used to assess effect size (0.01 = small, 0.06 = medium, 0.13 = large). The level of significance was set at *p* < 0.05, while for the testing of multiple univariate interaction effects, a Bonferroni adjustment was introduced by dividing the declared level of statistical significance by the number of dependent variables: *p* < 0.025 (i.e., *p* < 0.05 ÷ 2).

## 3. Results

Bivariate correlations of employed psychometric scales are shown in Table 1.

Significant associations resulted between self-efficacy and the three needs considered (information: −0.220 **, reassurance: −0.254 **, sharing: −0.185 **). The direction of the association was inverse; therefore, the increase in the perception of need correlated negatively with the perception of self-efficacy. Needs also showed strong correlations with respect to time orientation both in the present and in the future. In relation to anxieties, the perception of threat referred, in terms of time orientation, to a consistent concern for the present (0.324 **) and for the future (0.288 **). The activation of state anxiety showed a strong negative association with self-efficacy (−0.315 **), and at the same time was linked to a strong need for reassurance (0.281 **) and for sharing (0.207 **). There were no significant correlations of the measures with maternal age, while gestational age was positively associated with the need for reassurance (0.246 **) and negatively associated with a focus on the future (−0.226 **). 

In relation to the possible influence of socio-anagraphic variables on the study baseline measures, a preliminary ANOVA one-way analysis showed no significance of association (*p* > 0.05) of educational qualification and marital status on participants’ perceived needs, anxieties, self-efficacy, and temporal focus dimensions. On the other hand, the occupation/activity variable showed some significant differences: unemployed women showed a higher level of perceived threat (M = 2.08, SD = 0.93, *p* < 0.05) than the categories composed of factory workers (M = 1.00, SD = 0.21), office workers (M = 1.52, SD = 0.63), female students (M = 1.18, SD = 0. 12), self-employed (M = 1.50, SD = 0.64); female students showed a higher level of state anxiety (M = 4.40, SD = 0.39, *p* < 0.05) than the other categories composed of factory workers (M = 3.75, SD = 0.10), employed (M = 3.97, SD = 0.52), unemployed (M = 3.58, SD = 0.74), and self-employed (M = 4.06, SD = 0.40).

**Figure 1:**
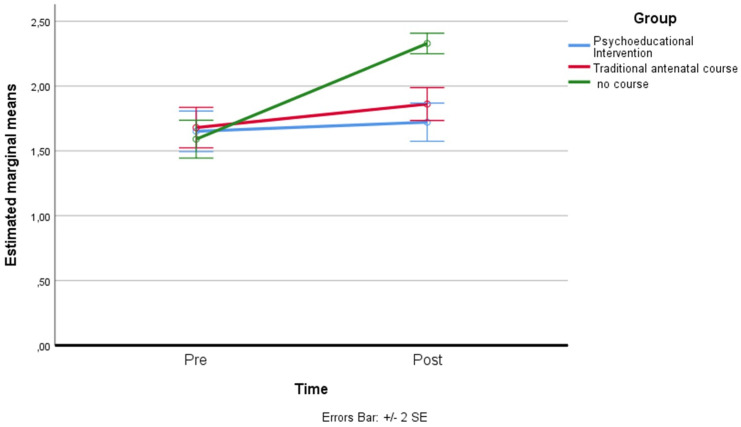
Estimated marginal means of threat anxiety.

*Perception related to Threat Anxiety*: a residual analysis was performed to test for the assumptions of the two-way repeated measures ANOVA. Outliers were assessed by inspection of a boxplot; normality was assessed using Shapiro–Wilk’s normality. Mauchly’s test of sphericity indicated that the assumption of sphericity was violated for the two-way interaction, χ^2^(2) = 63.75, *p* = 0.006, so the adaptation of Greenhouse–Geisser was considered. There was a statistically significant interaction between the psychoeducational intervention and time on threat anxiety (Emas P), F(1.283, 101.384) = 60.723, *p* < 0.001, partial η^2^ = 0.435. The main effect of time showed a statistically significant difference in mean treat anxiety at different time points: F(1, 79) = 162.061, *p* < 0.001, partial η^2^ = 0.672. The main effect of group showed that there was a statistically significant difference in mean threat anxiety between groups: F(1.714, 135.398) = 7.612, *p* < 0.05, partial η^2^ = 0.081. A post hoc analysis with a Bonferroni adjustment revealed that after two months, threat anxiety was statistically significantly greater in the control group (M = 2.33 ± 0.35) compared to the psychoeducational intervention group (M = 1.72 ± 0.66), *p* < 0.001 (see Figure 1). 

**Figure 2:**
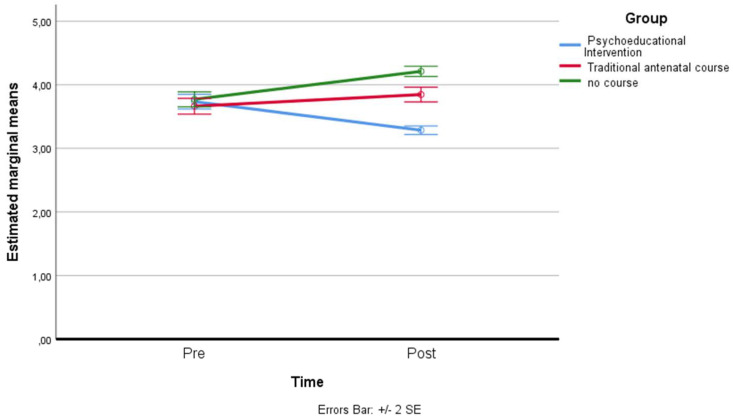
Estimated marginal means of state anxiety.

*Perception related to State Anxiety*: a residual analysis was performed to test for the assumptions of the two-way repeated measures ANOVA. Outliers were assessed by inspection of a boxplot; normality was assessed using Shapiro–Wilk’s normality. Mauchly’s test of sphericity indicated that the assumption of sphericity was violated for the two-way interaction, χ^2^(2) = 8.76, *p* = 0.012, so the adaptation of Greenhouse–Geisser was considered. There was a statistically significant interaction between the psychoeducational intervention and time on state anxiety (Emas-S), F(1.808, 142.819) = 71.076, *p* < 0.001, partial η^2^ = 0.474. The main effect of time did not show a statistically significant difference in mean state anxiety at different time points: F(1, 79) = 2.754, *p* > 0.05, partial η^2^ = 0.034. The main effect of group showed that there was a statistically significant difference in mean state anxiety between groups: F(1.742, 137.619) = 30.063, *p* < 0.001, partial η^2^ = 0.276. A post hoc analysis with a Bonferroni adjustment revealed that after two months, state anxiety was statistically significantly greater in the control group (M = 4.21 ± 0.36) compared to the traditional antenatal course (M = 3.85 ± 0.52), and to the psychoeducational intervention group (M = 3.28 ± 0.30), *p* < 0.001, while the latter showed a significantly lower level of state anxiety than that reported by the other two groups, *p* < 0.001 (see Figure 2). 

**Figure 3:**
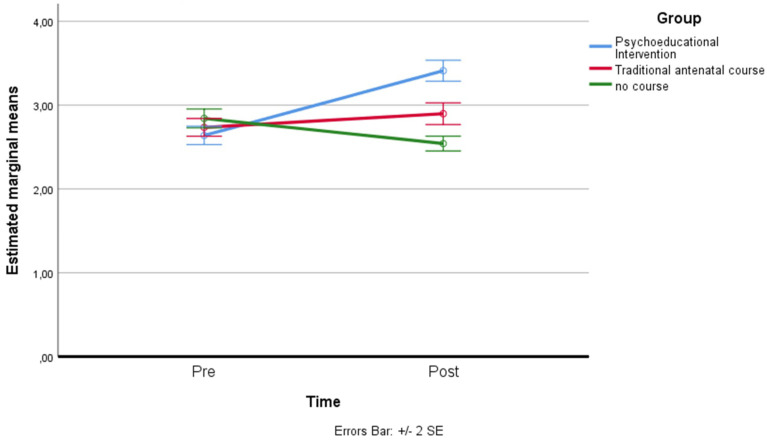
Estimated marginal means of perceived self-efficacy.

*Perceived Self-Efficacy:* a residual analysis was performed to test for the assumptions of the two-way repeated measures ANOVA. Outliers were assessed by inspection of a boxplot; normality was assessed using Shapiro–Wilk’s normality. Mauchly’s test of sphericity indicated that the assumption of sphericity was not violated for the two-way interaction: χ^2^(2) = 2.17, *p* = 0.337. There was a statistically significant interaction between the psychoeducational intervention and the time on perceived self-efficacy: F(2, 158) = 43.152, *p* < 0.001, partial η^2^ = 0.353. The main effect of time showed a statistically significant difference in mean self-efficacy at different time points: F(1, 79) = 25.004, *p* < 0.001, partial η^2^ = 0.240. The main effect of the group showed that there was a statistically significant difference in mean perceived self-efficacy between groups: F(2, 158) = 18.444, *p* < 0.001, partial η^2^ = 0.189. A post hoc analysis with a Bonferroni adjustment revealed that after two months, perceived self-efficacy was statistically significantly greater in the psychoeducational intervention group (M = 3.41 ± 0.56) compared to the control group (M = 2.54 ± 0.39), and to the traditional antenatal course (M = 2.90 ± 0.58), *p* < 0.001 (see Figure 3). 

**Figure 4:**
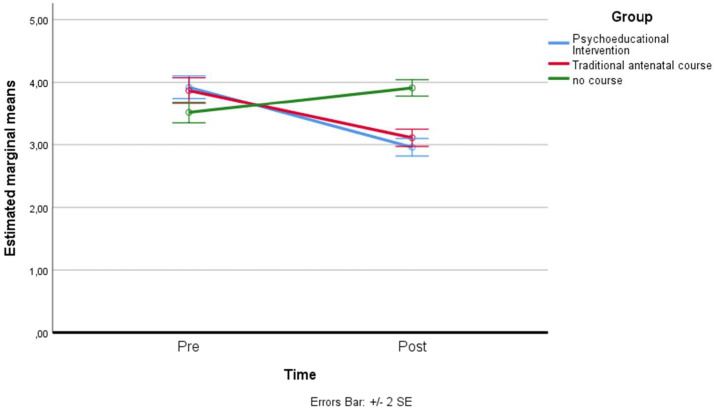
Estimated marginal means of perceived need for information.

*Perceived Need for Information:* a residual analysis was performed to test for the assumptions of the two-way repeated measures ANOVA. Outliers were assessed by inspection of a boxplot; normality was assessed using Shapiro–Wilk’s normality. Mauchly’s test of sphericity indicated that the assumption of sphericity was not violated for the two-way interaction: χ^2^(2) = 0.191, *p* = 0.998. There was a statistically significant interaction between the psychoeducational intervention and time on the perceived need for information: F(2, 158) = 40.412, *p* < 0.001, partial η^2^ = 0.338. The main effect of time showed a statistically significant difference in mean perceived need for information at different time points: F(1, 79) = 48.922, *p* < 0.001, partial η^2^ = 0.382. The main effect of the group showed that there was a statistically significant difference in mean perceived need for information between groups: F(2, 158) = 5.669, *p* < 0.01, partial η^2^ = 0.067. A post hoc analysis with a Bonferroni adjustment revealed that after two months, the perceived need for information was statistically significantly greater in the control group (M = 3.91 ± 0.59) compared to the traditional antenatal group (M = 3.11 ± 0.61), and to the psychoeducational intervention course group (M = 2.96 ± 0.62), *p* < 0.001 (see Figure 4). 

**Figure 5:**
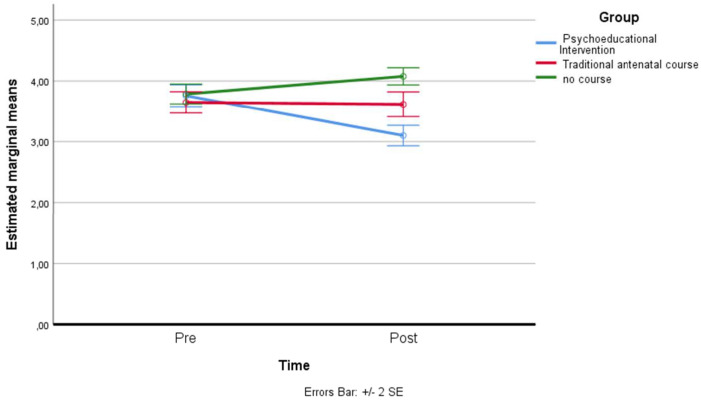
Estimated marginal means of perceived need for reassurance.

*Perceived Need for Reassurance*: a residual analysis was performed to test for the assumptions of the two-way repeated measures ANOVA. Outliers were assessed by inspection of a boxplot; normality was assessed using Shapiro–Wilk’s normality. Mauchly’s test of sphericity indicated that the assumption of sphericity was not violated for the two-way interaction: χ^2^(2) = 1.38, *p* = 0.501. There was a statistically significant interaction between the psychoeducational intervention and time on the perceived need for reassurance: F(2, 158) = 19.794, *p* < 0.001, partial η^2^ = 0.200. The main effect of time showed a statistically significant difference in mean perceived need for reassurance at different time points: F(1, 79) = 4.837, *p* < 0.05, partial η^2^ = 0.058. The main effect of the group showed that there was a statistically significant difference in mean perceived need for reassurance between groups: F(2, 158) = 12.584, *p* < 0.001, partial η^2^ = 0.137. A post hoc analysis with a Bonferroni adjustment revealed that after two months, the perceived need for reassurance results were statistically significantly greater in the control group (M = 4.08 ± 0.63) compared to the traditional antenatal group (M = 3.62 ± 0.92), and to the psychoeducational intervention course group (M = 3.10 ± 0.75), while the latter was significantly lower than those of the other two groups, *p* < 0.001 (see Figure 5). 

**Figure 6:**
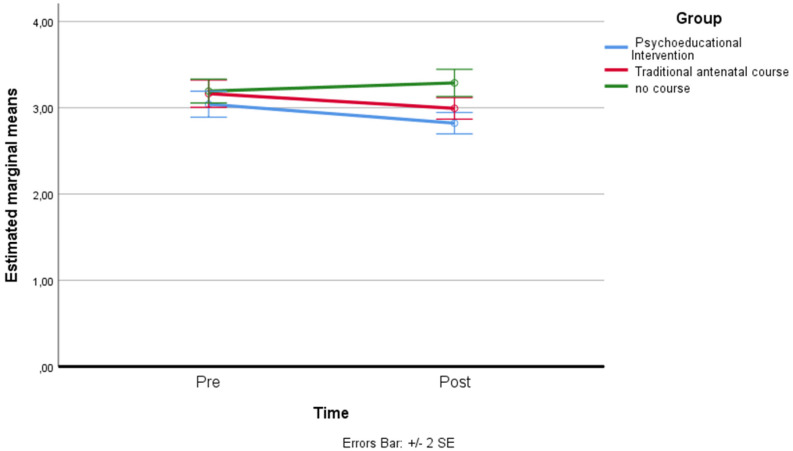
Estimated marginal means of perceived need for sharing.

*Perceived Need for Sharing*: a residual analysis was performed to test for the assumptions of the two-way repeated measures ANOVA. Outliers were assessed by inspection of a boxplot; normality was assessed using Shapiro–Wilk’s normality. Mauchly’s test of sphericity indicated that the assumption of sphericity was not violated for the two-way interaction: χ^2^(2) = 2.79, *p* = 0.249. There was a statistically significant interaction between the psychoeducational intervention and time on the perceived need for sharing: F(2, 158) = 3.534, *p* < 0.05, partial η^2^ = 0.043. The main effect of time did not show a statistically significant difference in the mean perceived need for sharing at different time points: F(1, 79) = 3.292, *p* > 0.05, partial η^2^ = 0.040. The main effect of the group showed that there was a statistically significant difference in the mean perceived need for sharing between groups: F(2, 158) = 7.695, *p* < 0.05, partial η^2^ = 0.089. A post hoc analysis with a Bonferroni adjustment revealed that after two months, the perceived need for sharing was statistically significantly greater in the control group (M = 3.29 ± 0.70) compared to the traditional antenatal group (M = 2.99 ± 0.56), and to the psychoeducational intervention course group (M = 2.82 ± 0.55), *p* < 0.001 (see Figure 6). 

**Figure 7:**
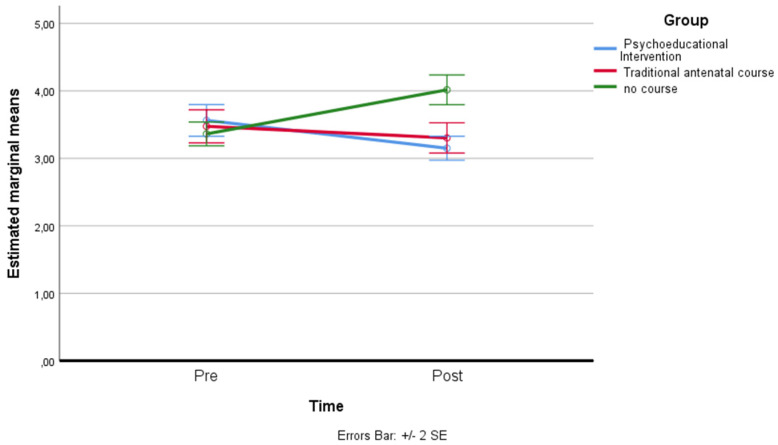
Estimated marginal means of past focus.

*Past focus*: a residual analysis was performed to test for the assumptions of the two-way repeated measures ANOVA. Outliers were assessed by inspection of a boxplot; normality was assessed using Shapiro–Wilk’s normality. Mauchly’s test of sphericity indicated that the assumption of sphericity was violated for the two-way interaction; therefore, the Greenhouse–Geisser adaptation was considered: χ^2^(2) = 6.953, *p* = 0.031. There was a statistically significant interaction between the psychoeducational intervention and time on past focus: F(1.843, 145.585) = 15.279, *p* < 0.001, partial η^2^ = 0.162. The main effect of time did not show a statistically significant difference in mean past focus at different time points: F(1, 79) = 0.093, *p* > 0.05, partial η^2^ = 0.001. The main effect of the group showed that there was a statistically significant difference in mean past focus between groups: F(2, 158) = 4.751, *p* < 0.05, partial η^2^ = 0.057. A post hoc analysis with a Bonferroni adjustment revealed that after two months, past focus was statistically significantly greater in the control group (M = 4.01 ± 0.98) compared to the traditional antenatal group (M = 3.30 ± 1.00), and to the psychoeducational intervention course group (M = 3.15 ± 0.79), *p* < 0.001 (see Figure 7). 

**Figure 8:**
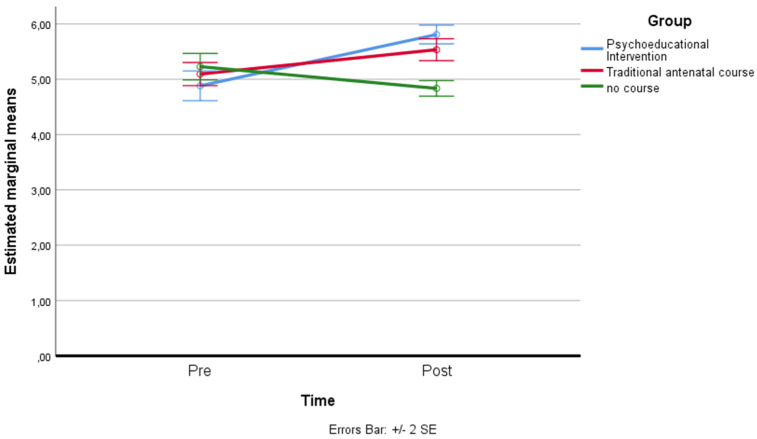
Estimated marginal means of present focus.

*Present focus*: a residual analysis was performed to test for the assumptions of the two-way repeated measures ANOVA. Outliers were assessed by inspection of a boxplot; normality was assessed using Shapiro–Wilk’s normality. Mauchly’s test of sphericity indicated that the assumption of sphericity was not violated for the two-way interaction: χ^2^(2) = 4.339, *p* = 0.114. There was a statistically significant interaction between the psychoeducational intervention and time on present focus: F(2, 158) = 27.292, *p* < 0.001, partial η^2^ = 0.257. The main effect of time showed a statistically significant difference in mean present focus at different time points: F(1, 79) = 16.525, *p* < 0.001, partial η^2^ = 0.173. The main effect of the group showed that there was a statistically significant difference in mean present focus between groups: F(2, 158) = 4.647, *p* < 0.05, partial η^2^ = 0.056. A post hoc analysis with a Bonferroni adjustment revealed that after two months, present focus was statistically significantly lower in the control group (M = 4.83 ± 0.63) compared to the traditional antenatal group (M = 5.53 ± 0.88), and to the psychoeducational intervention course group (M = 5.81 ± 0.76), *p* < 0.001 (see Figure 8). 

**Figure 9:**
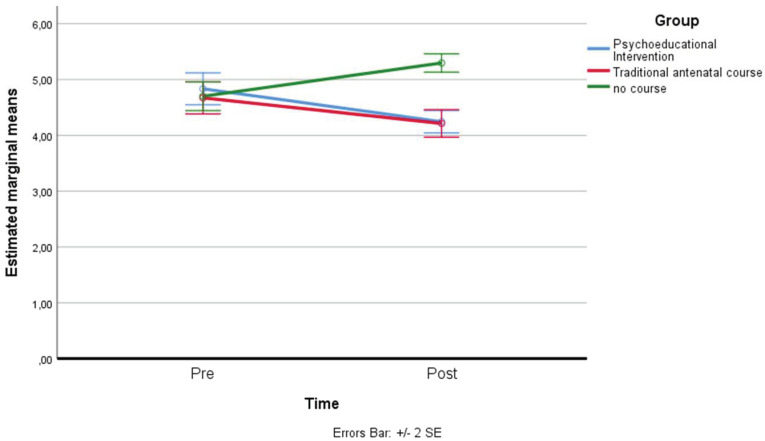
Estimated marginal means of future focus.

*Future focus*: a residual analysis was performed to test for the assumptions of the two-way repeated measures ANOVA. Outliers were assessed by inspection of a boxplot; normality was assessed using Shapiro–Wilk’s normality. Mauchly’s test of sphericity indicated that the assumption of sphericity was not violated for the two-way interaction: χ^2^(2) = 5.096, *p* = 0.078. There was a statistically significant interaction between the psychoeducational intervention and time on future focus: F(2, 158) = 13.042, *p* < 0.001, partial η^2^ = 0.142. The main effect of time did not show a statistically significant difference in mean future focus at different time points: F(1, 79) = 2.820, *p* > 0.05, partial η^2^ = 0.034. The main effect of the group showed that there was a statistically significant difference in mean future focus between groups: F(2, 158) = 12.676, *p* < 0.001, partial η^2^ = 0.138. A post hoc analysis with a Bonferroni adjustment revealed that after two months, future focus was statistically significantly greater in the control group (M = 5.30 ± 0.74) compared to the traditional antenatal group (M = 4.21 ± 1.10), and to the psychoeducational intervention course group (M = 4.24 ± 0.89), *p* < 0.001 (see Figure 9). 

Table 2 below summarizes the significant differences obtained in the pre and post measurement comparing the three groups of participating women.

## 4. Discussion

To the best of our knowledge, this study presents one of the few prepartum training programs with an extensive duration (eight out of fifteen meetings) devoted to aspects of the psychological well-being and mental health of pregnant women. This approach arose from the consideration of both the relevance of mental health problems that can occur during pregnancy and after childbirth, and the limitations of traditional programs reported in the literature which, while offering useful knowledge and skills to pregnant women and the family, generally fail to address within the groups issues that correspond to relevant needs related to the management of emotions, in particular anxiety and fear of childbirth, psychological preparation for the reception of the newborn, acceptance of bodily changes and lifestyle, and the couple’s healthy management of the new motherhood/fatherhood [68,69,71,85].

The results obtained from our study showed the effectiveness of the psychoeducational intervention on all nine measures considered and collected before and after a two-month interval, corresponding to the end of the classes of the course with psychoeducational content enrichment and the traditional prepartum course. While measurements at baseline reported no significant differences between groups, significant effects emerged after the interval on most of the variables targeted by the intervention. 

With regard to anxiety, the perception of threat seems to have been well contained by the provision of the two prepartum courses, both the one with psychoeducational characterization and the traditional basic one. In contrast, in the control group there was a significant increase in this fear after two months. For state anxiety, the psychoeducational intervention showed a clear efficacy both compared to the control group and to the group that had attended the traditional course; although, the latter showed lower levels of anxiety than the control group. 

Equally relevant were the effects of the psychoeducational intervention on perceived self-efficacy. Participants in the psychoeducational group reported significantly higher self-efficacy values than those in the basic course group and the control group, where the lowest levels of perceived self-efficacy were reported for the latter. 

In the needs assessment, it emerged that the conclusion of the two courses corresponded to a significant decrease in the need for information, which instead showed a substantial increase in the control group. The psychoeducational intervention showed a significant decrease in the need for reassurance, which for those attending the traditional course remained unchanged, while it showed an increase in the control group. With regard to the need for sharing, the two courses both showed that they could satisfy this need, while for the control group this need continued to be present in the course of time. 

With regard to the temporal focus, it could be observed that while in those who had attended the prenatal courses there was an increase in their focus on the present time, in the control group there was a significant shift in the focus of women in labor towards the past and the future. Associations of such shifts in focus with increased anxiety and insecurity have been reported in the literature [86,87]. The general picture appears further clarified by the low self-efficacy perceived by women who had not attended any course, depicting a group of parturients for whom the most threatening component of their anxiety was associated with a projection of the worry in the future (given the perception of threat caused by the unknown of childbirth and its potential complications). This crucial dimension of lack of control over a condition which is experienced as threatening for the person, as also reported in Wigert et al. [70], was also reflected in the negative association of this component of anxiety with perceived self-efficacy. 

Women who had attended the course with psychoeducational characterization tended to be more concentrated on the present, while thoughts directed to the past and focusing on the future were significantly reduced. Focus on the present also corresponded to a simultaneous decrease in state anxiety. In operational terms, this can suggest that it is preferable to refocus attention on the present, avoiding both anticipatory but uncontrollable projections, and defensive compensations on the past, in order to get a good management of the ongoing process [88,89].

The present study suggests taking due account of the significant association between perinatal anxiety and the parturient’s need for information, reassurance, and sharing. The usually limited size of prenatal groups can be an important element, as it allows for interaction between members and the exchange of meaningful experiences, the circulation and processing of information received, and the construction of networks and significant links between participants and staff, contributing to a climate of greater reassurance. Concern for the future is accompanied by an increased tendency for women to share their own experiences, critical emotions, doubts, etc., with other women. Therefore, for the parturients, the group appears to be an elective place for the collection of reliable information and for regaining control in the present, thus producing greater reassurance globally. These considerations are also in line with Brixval et al. [57] on the desirability of promoting greater self-efficacy in childbirth through interventions with small groups of parturients.

Several studies have highlighted the beneficial function of self-efficacy in parturients, and the desirability of measures to strengthen it [90,91,92]. Carlsson et al. [93] showed that childbirth self-efficacy was correlated with positive dimensions such as vigor, sense of coherence, and maternal support and negatively correlated with previous mental illness, negative mood states, and fear of childbirth. Women who reported high childbirth self-efficacy had less epidural analgesia during childbirth, compared to women with low self-efficacy. Childbirth self-efficacy is therefore a positive dimension that interplays with other aspects and contributes to well-being during pregnancy, and thereby acts as an asset in the context of childbirth. Further studies [94,95] reported that increased childbirth self-efficacy is associated with a wide variety of improved perinatal outcomes. Moreover, according to these, there is evidence that childbirth self-efficacy is a psychosocial factor that can be modified through various efficacy-enhancing interventions. 

In order to achieve this objective, the communication style adopted within the groups and the dynamics of interpersonal responsiveness are also relevant [96,97,98,99]. In the study by Ho and Holroyd [100], it was reported that women often identified that it was impossible to engage in questioning and discussion, to have their personal problems addressed, to make friends, and for the educators to seek appropriate feedback from their clients. Women do not attend antenatal classes solely to receive information and develop skills. The social agenda, namely, the opportunity to meet other women experiencing pregnancy at the same time, is also a high priority [101].

Keeping in mind these needs, both reported in the literature and better identified through the construction of the SNEP scale used in this study along with other instruments administered to pregnant women, the teaching methodology proposed in the psychoeducational intervention recognized the active involvement of pregnant women, inviting them to freely report experiences, emotions, expectations, fears, discomfort, and any uncertainties in the group. 

The course focused on the acquisition of a greater awareness of the situation and on the possibility of exercising positive control starting from daily routines. The choice and inclusion of pleasant activities in the planning of the day, for example, was aimed at achieving greater balance and psychophysical well-being, balancing the pressures and conditioning perceived in the advancement of the pregnancy process. The learning acquired through the development of relaxation techniques in the group and the application of strategies for stress modulation provided them not only with a pleasant sharing interval, which presumably contributed to an increase group cohesion, but above all with useful tools that could be employed later (even after childbirth) for a functional approach to cope with moments of greater stress and emotional difficulty. Moreover, the reflection on the importance of communication and on a correct use of expressive tools had the objective of promoting balanced and functional interactions within the couple; channeling in a constructive way the expression of needs, frustrations, and fears; and restraining potential conflicts and misunderstandings with the partner. In the general content plan, it seemed appropriate to emphasize the reflection on the conditioning of cultural models related to motherhood and the role of mother, starting from the family or the surrounding environment of reference. This was with a view to a mature understanding of these influences in order to consciously reach an acceptance or a reasonable rejection of the expected role, in favor of alternative models of motherhood more congruent with their sensitivity and with the development of their own identity image. Finally, the incentive to write and keep a diary of daily actions, thoughts, and emotions recurring during the period approaching childbirth was aimed at continuing individually the work of active elaboration of the process of transition to motherhood and ensuring further control and monitoring of processes in progress, through the recording of highlights, positive changes, and the anticipation of future plans. Considering the results in our study, it was found that the psychoeducational intervention has actually induced significant and positive changes especially with regard to four dimensions: state anxiety, perceived self-efficacy, the need for information, and reassurance on the part of the pregnant women who participated in this experiment. The evidence collected suggests that the intervention protocol described should be replicated further, in order to improve the psychophysical well-being of women in a delicate moment such as pregnancy and preparation for childbirth, but especially in terms of prevention and containment of the risks of psychological distress that frequently affect a significant number of women after childbirth.

## 5. Limitations and Suggestions

It is also worth noting among the limitations of this study that the sample of the participants was small; the time interval between the test and the retest was rather close (two months). It would also be beneficial to have a follow-up monitoring after childbirth, in order to collect ex post evaluations on the effectiveness of the notions and skills acquired during the course. In addition, it is recommended that other lifestyle factors be included that have been shown to influence psychological dimensions, such as physical exercise and sleep quality [102,103]. Further studies could also test whether and to what extent the relationships between self-efficacy and anxiety are mediated by women’s needs and expectations. It should also be considered that the protocol did not include a specific measure of fear of childbirth, so it was not possible to verify whether this increase in self-efficacy was associated with a decrease in fear of childbirth as well; however, the results reported an interesting association with a decrease in state anxiety. Finally, it should be kept in mind that the intervention group had a different “dose” of classes than the traditional course group: the latter had 2 h classes once a week for 8 weeks, while the intervention group had 2 h classes twice a week for 8 weeks; therefore, they had twice as much support from professionals and twice as much contact/support from other expectant parents. This may have contributed to some of the benefits and should be acknowledged.

## 6. Conclusions

The most innovative aspect of this work was the implementation of a childbirth preparation program which included a very extensive section of content regarding the psychological well-being and mental health of pregnant women. In this way, a specific gap in current prenatal training was thought to have been filled. The study also emphasized the association between the time perspective of pregnant women and the specific anxieties they bear. In this respect, a measure that identifies and evaluates the individual perception of basic needs activated in the condition of pregnancy (i.e., the need for information, sharing, reassurance) was contextually constructed and administered. The psychoeducational program used methods of active involvement and group learning of relaxation techniques and stress control; it also promoted the practice of assertive communication, monitoring, and control of routines through the use of a diary; it promoted mental well-being through the use of techniques of cognitive restructuring and emotional regulation; and it increased levels of awareness through a shared reflection on the processes of physical transformation, role transition, and cultural conditioning of models of motherhood. The comparison (pre–post) with a group of pregnant women attending a traditional course and with a control group of pregnant women who did not attend any course showed that the psychoeducational intervention prompted, at its conclusion, a higher level of perceived self-efficacy and significantly reduced the levels of anxiety by directing the temporal focus of participants to the functional management of the present. A possible continuation of the study could also provide follow-up indications on the effectiveness of such a program in preventing the forms of psychological and relational discomfort that can occur after childbirth.

## Figures and Tables

**Table 1:** Bivariate correlations.

	MA	GA	IN	RN	SN	PSE	Past	Pres	Fut	PT	SA
MA	1										
GA	−0.105	1									
IN	0.026	0.147	1								
RN	−0.001	0.246 **	0.685 **	1							
SN	−0.028	−0.074	0.431 **	0.468 **	1						
PSE	−0.045	0.097	−0.220 **	−0.254 **	−0.185 **	1					
Past	0.032	0.021	−0.066	0.012	0.062	−0.078	1				
Pre	0.124	−0.052	0.281 **	0.303 **	0.292 **	0.024	−0.078	1			
Fut	0.142	−0.226 **	0.206 **	0.252 **	0.341 **	−0.045	0.340 **	0.370 **	1		
TA	−0.106	−0.001	0.106	0.281 **	0.207 **	−0.315 **	0.063	−0.068	0.252 **	1	
SA	−0.003	0.082	0.135	0.211 **	0.144 *	−0.122	0.046	0.324 **	0.288 **	−0.003	1

Note: MA = Maternal Age; GA = Gestational Age (weeks); IN = Information Need; RN = Reassurance Need; SN = Sharing Need; PSE = Perceived Self-Efficacy; Past = Past Focus; Pre = Present Focus; Fut = Future Focus; TA = Threat Anxiety; SA = State Anxiety. N = 102; ** correlation is significant at *p* < 0.005 2-tailed; * correlation is significant at *p* < 0.001 2-tailed. Spearman’s coefficient was used for correlations with maternal and gestational age, while associations between the remaining variables were performed through Pearson’s coefficient.

**Table 2:** Significant differences in the measurements between the three groups.

		Psychoeducational Intervention Group (*n* = 80)	Traditional Antenatal Course Group (*n* = 80)	No Course Group (*n* = 80)			
Variables	Time	(Mean ± SD)	(Mean ± SD)	(Mean ± SD)	F	*p*-Value	η^2^
Threat Anxiety	PRE	1.65 ± 0.70	1.67 ± 0.69	1.59 ± 0.65	0.356	>0.05	0.003
	POST	1.72 ± 0.65	1.86 ± 0.56	2.32 ± 0.35	27.592	<0.001	0.189
State Anxiety	PRE	3.73 ± 0.52	3.66 ± 0.56	3.77 ± 0.53	0.858	>0.05	0.007
	POST	3.29 ± 0.30	3.85 ± 0.52	4.21 ± 0.36	107.038	<0.001	0.475
Self-Efficacy	PRE	2.64 ± 0.49	2.74 ± 0.47	2.84 ± 0.50	3.450	>0.05	0.028
	POST	3.41 ± 0.56	2.90 ± 0.58	2.54 ± 0.39	57.157	<0.001	0.325
Information Need	PRE	3.92 ± 0.81	3.87 ± 0.92	3.51 ± 0.75	5.728	>0.05	0.046
	POST	2.96 ± 0.63	3.11 ± 0.61	3.91 ± 0.59	56.707	<0.001	0.324
Reassurance Need	PRE	3.76 ± 0.80	3.65 ± 0.78	3.79 ± 0.73	0.712	>0.05	0.006
	POST	3.10 ± 0.75	3.62 ± 0.92	4.08 ± 0.63	31.666	<0.001	0.211
Sharing Need	PRE	3.04 ± 0.67	3.16 ± 0.71	3.19 ± 0.61	1.169	>0.05	0.010
	POST	2.82 ± 0.55	2.99 ± 0.56	3.29 ± 0.67	12.620	<0.001	0.096
Past Focus	PRE	3.56 ± 1.05	3.47 ± 1.09	3.36 ± 0.79	0.825	>0.05	0.007
	POST	3.15 ± 0.79	3.30 ± 1.00	4.01 ± 0.98	19.610	<0.001	0.142
Present Focus	PRE	4.88 ± 1.20	5.09 ± 0.93	5.23 ± 1.07	2.119	>0.05	0.018
	POST	5.81 ± 0.76	5.53 ± 0.88	4.83 ± 0.63	34.534	<0.001	0.226
Future Focus	PRE	4.83 ± 1.28	4.67 ± 1.29	4.70 ± 1.15	0.392	>0.05	0.003
	POST	4.24 ± 0.89	4.21 ± 1.10	5.30 ± 0.74	35.689	<0.001	0.231

## Data Availability

The datasets during and/or analyzed during the current study are available from the corresponding author on reasonable request.
